# Supplementary material for: A Systematic Review of Areal Units and Adjacency Used in Bayesian Spatial and Spatio-Temporal Conditional Autoregressive Models in Health Research
Source: Int J Environ Res Public Health. 2023 Jul 1;20(13):6277. doi: 10.3390/ijerph20136277 (PMC10341419; doi:10.3390/ijerph20136277)
Supplement: Supplementary file 1 [file ijerph-20-06277-s001.zip › Table S1.pdf]

Table S1: Searching terms used in each database.

Databases	Search terms
**Medline**	(Bayesian [MeSH Terms] AND spatial [MeSH Terms] OR spatialtemporal [MeSH Terms] OR spatial-temporal [MeSH Terms] OR spatiotemporal [MeSH Terms] OR spatio-temporal [MeSH Terms] OR space-time[MeSH Terms] OR geo-temporal model [MeSH Terms] OR geotemporal model [MeSH Terms] geographic-temporal model [MeSH Terms] AND conditional autoregressive[MeSH Terms]) AND (("2012/01/01"[Date - Publication] : "2022/09/08"[Date - Publication]))
**Embase**	(Bayesian.mp AND spatial.mp OR spatialtemporal.mp OR spatial-temporal.mp OR spatio-temporal.mp OR spatiotemporal*.mp OR space-time*.mp OR geo-temporal.mp OR geotemporal.mp OR geographic temporal AND Conditional autoregressive.mp AND (("2012/01/01"[Date - Publication] : "2022/09/08"[Date - Publication]))
**PschINFO**	(Bayesian.mp AND spatial.mp OR spatialtemporal.mp OR spatial-temporal.mp OR spatiotemporal.mp OR spatio-temporal.mp *.mp OR space-time model*.mp OR geo-temporal.mp OR geotemporal.mp OR geographic-temporal AND conditional autoregressive.mp) AND (("2012/01/01"[Date - Publication]: "2022/09/08"[Date - Publication]))
**PubMed**	(Bayesian [MeSH Terms])) AND (spatial[MeSH Terms])) OR (spatialtemporal[MeSH Terms])) OR (spatial-temporal[MeSH Terms])) OR (spatiotemporal[MeSH Terms])) OR (spatio-temporal[MeSH Terms])) OR (space-time[MeSH Terms])) OR (geo-temporal[MeSH Terms])) OR (geotemporal[MeSH Terms])) OR (geographic-temporal[MeSH Terms])) AND (Conditional autoregressive[MeSH Terms]) AND (("2012/01/01"[Date -Publication] : "2022/09/08"[Date - Publication]))
**SCOPUS**	TITLE-ABS-KEY (Bayesian) AND TITLE-ABS-KEY (spatial) OR TITLE-ABS-KEY (spatialtemporal) OR TITLE-ABS-KEY (spatial-temporal) OR TITLE-ABS-KEY (spatiotemporal) OR TITLE-ABS-KEY (spatio-temporal) OR TITLE-ABS-KEY (space-time OR TITLE-ABS-KEY (geo-temporal) OR (TITLE-ABS-KEY (geographic-temporal AND

	TITLE-ABS-KEY(conditional autoregressive)) AND (("2012/01/01"[Date -Publication] : "2022/09/08"[Date - Publication]))
**Web of Science**	(TS=(Bayesian)) AND TS=(Spatial)) OR TS=(spatialtemporal)) OR TS=(spatial-temporal)) OR TS=(spatiotemporal)) OR TS=(spatio-temporal)) OR ALL=(space-time)) OR TS=(geo-temporal)) OR TS=(geotemporal)) OR TS=(geographic-temporal)) AND TS=(Conditional autoregressive) AND (("2012/01/01"[Date -Publication] : "2022/09/08"[Date - Publication]))
**Emcare**	(Bayesian.mp AND spatial.mp OR spatialtemporal.mp OR spatial-temporal.mp OR spatiotemporal.mp OR spatio-temporal.mp OR space-time.mp OR geo-temporal.mp OR geo-temporal.mp OR geographic-temporal AND conditional autoregssive.mp) AND ("2012/01/01"[Date - Publication]: "2022/09/08"[Date - Publication]))
