# Supplementary material for: A Systematic Review of Areal Units and Adjacency Used in Bayesian Spatial and Spatio-Temporal Conditional Autoregressive Models in Health Research
Source: Int J Environ Res Public Health. 2023 Jul 1;20(13):6277. doi: 10.3390/ijerph20136277 (PMC10341419; doi:10.3390/ijerph20136277)
Supplement: Supplementary file 1 [file ijerph-20-06277-s001.zip › Table S2.pdf]

Table S2: Risk of bias tool for assessment

ID	Author	Year	AaO	SaP	MS	MM	PRD	QoD	PoR	IDOR	Sum	Rating
1	Adeyemi et al. 2019	2019	2	2	1	2	2	2	1	1	13	High
2	Akter et al. 2021	2021	2	2	2	2	2	2	2	2	15	Very high
3	Alam et al. 2019	2019	1	2	1	1	2	1	1	2	12	High
4	Alene et al. 2021	2021	2	2	1	2	2	1	1	2	13	High
5	Amsalu et al. 2019	2019	1	1	1	1	1	1	1	2	9	Medium
6	Aragonés et al. 2013	2013	2	2	1	1	2	1	1	2	12	High
7	Aswi et al.2020	2020	2	2	2	1	2	0	2	1	12	High
8	Aswi et al.2020	2020	1	1	1	1	2	2	1	0	9	Medium
9	Baker et al.2017	2017	2	1	2	1	2	2	1	1	14	Very high
10	Blain et al. 2013	2013	2	2	1	1	2	1	2	1	12	High
11	Chou et al.2014	2014	2	2	2	1	2	2	2	2	15	Very high
12	M. Cramb et al.2015	2015	2	2	1	1	2	1	2	1	12	High
13	Danwang et al.2021	2021	2	2	2	2	2	1	2	2	15	Very high
14	Darikwa et al.2020	2020	2	2	1	1	2	2	1	2	13	High
15	Desjardinset al. 2020	2020	2	2	1	1	2	2	1	2	13	High
16	Dhewantara et al. 2019	2019	2	2	2	2	1	2	2	2	16	Very high
17	Donkor et al. 2021	2021	2	2	1	1	1	1	1	1	10	Medium
18	Feng et al.2015	2015	2	2	1	1	1	2	1	1	11	High
19	Gelaw et al.2019	2019	2	2	2	2	2	2	2	2	16	Very high
20	Hanandita et al. 2016	2016	2	2	1	2	2	2	1	1	13	High
21	Hu et al. 2012	2012	1	2	2	1	1	1	1	1	10	Medium
22	Huang et al.2017	2017	1	1	2	2	1	2	1	1	12	High
23	Ibeji et al. 2022	2022	2	2	2	2	2	2	1	1	14	Very high
24	Jurgens et al. 2013	2017	2	2	2	2	2	2	2	1	15	Very high
25	Kandhasamy et al. 2017	2017	2	2	1	2	2	1	1	1	12	High
26	Kigozi et al.2020	2020	2	1	1	2	2	2	2	1	13	High

27	Lal et al. 2020	2020	2	2	2	2	2	2	2	1	15	Very high
28	Law. 2016	2016	2	2	2	2	2	2	2	1	15	Very high
29	Li et al. 2020	2020	2	2	1	2	2	1	1	1	12	High
30	Lubinda et al. 2021	2021	2	2	2	2	1	2	1	1	13	High
31	Lome-Hurtado et al.2021	2021	2	2	2	2	2	2	2	2	14	Very high
32	Lome-Hurtado et al.2021	2021	2	2	1	1	2	1	1	1	11	High
33	Ngwira. 2022	2022	2	2	1	1	2	2	1	1	12	High
34	Ntirampeb et al.2018	2018	1	1	1	1	1	1	1	1	8	Medium
35	Odhiambo et al. 2020	2020	2	2	1	1	2	2	1	1	12	High
36	Ogunsakin et al. 2022	2022	2	2	1	1	2	2	1	1	12	High
37	Okango et al.2015	2015	1	2	1	1	1	1	1	1	9	Medium
38	Okango et al.2016	2016	2	1	2	1	1	1	1	1	8	Medium
39	Okunlola et al, 2021	2021	2	2	1	1	1	1	1	1	10	Medium
40	Qi et al. 2014	2014	1	1	1	1	1	1	1	1	8	Medium
41	Raei et al.2018	2018	1	1	1	1	1	1	1	1	8	Medium
42	Reid et al. 2012	2012	2	2	1	2	2	2	1	1	13	High
43	Roza et al.2012	2012	2	2	2	2	2	2	2	2	16	Very high
44	Saijo et al.2018	2018	2	2	1	1	2	2	1	2	13	High
45	Sharafi et al.2018	2018	2	2	1	2	2	2	2	2	15	Very high
46	Thiam et al. 2019	2019	2	2	1	1	2	1	1	2	12	High
47	Tsheten el al, 2020	2020	2	2	1	2	2	1	1	2	13	High
48	Wangdi et al. 2017	2017	1	1	1	1	1	1	1	2	9	Medium
49	Wangdi et al. 2018	2018	2	2	1	1	2	1	1	2	12	High
50	Wangdi et al. 2022	2022	2	2	2	1	2	0	2	1	12	High
51	Wangdi et al.2020	2020	1	1	1	1	2	2	1	0	9	Medium
52	Xu et al. 2015	2015	2	2	2	1	2	2	2	1	14	Very high
Range			1-2	1-2	1-2	1-2	1-2	0-2	1-2	0-2	8-16	
Median score			2	2	1	1	2	2	1	1	12	high
Mean score			1.7	1.8	1.3	1.4	1.7	1.5	1.3	1.3	12.2	

AaO, aims and objectives; SaP, setting and population; MS, model structure; MM, modelling methods; PRDS, parameter ranges and data sources; QoD, quality of data; PoR, presentation of results; IDoR, interpretation, and discussion of results
