# Supplementary material for: A Systematic Review of Areal Units and Adjacency Used in Bayesian Spatial and Spatio-Temporal Conditional Autoregressive Models in Health Research
Source: Int J Environ Res Public Health. 2023 Jul 1;20(13):6277. doi: 10.3390/ijerph20136277 (PMC10341419; doi:10.3390/ijerph20136277)
Supplement: Supplementary file 1 [file ijerph-20-06277-s001.zip › Table S3.pdf]

Table S3: - List of publications included for systematic review

ID	Title
1	Adeyemi RA, Zewotir T, Ramroop S. Joint spatial mapping of childhood anemia and malnutrition in sub-Saharan Africa: a cross-sectional study of small-scale geographical disparities. African health sciences. 2019 Nov 7;19(3):2692-712.
2	Akter R, Hu W, Gatton M, Bambrick H, Cheng J, Tong S. Climate variability, socio-ecological factors and dengue transmission in tropical Queensland, Australia: A Bayesian spatial analysis. Environmental Research. 2021 Apr 1;195:110285.
3	Alam MS, Hossain SS, Sheela FF. Spatial smoothing of low birth weight rate in Bangladesh using Bayesian hierarchical model. Journal of Applied Statistics. 2019 Jul 27;46(10):1870-85.
4	Alene KA, Xu Z, Bai L, Yi H, Tan Y, Gray DJ, Viney K, Clements AC. Spatiotemporal Patterns of Tuberculosis in Hunan Province, China. International journal of environmental research and public health. 2021 Jun 24;18(13):6778.
5	Amsalu E, Liu M, Li Q, Wang X, Tao L, Liu X, Luo Y, Yang X, Zhang Y, Li W, Li X. Spatial-temporal analysis of tuberculosis in the geriatric population of China: An analysis based on the Bayesian conditional autoregressive model. Archives of gerontology and geriatrics. 2019 Jul 1;83:328-37.
6	Aragonés N, Goicoa T, Pollán M, Militino AF, Pérez-Gómez B, López-Abente G, Ugarte MD. Spatio-temporal trends in gastric cancer mortality in Spain: 1975–2008. Cancer epidemiology. 2013 Aug 1;37(4):360-9.
7	Aswi A, Cramb S, Duncan E, Hu W, White G, Mengersen K. Climate variability and dengue fever in Makassar, Indonesia: Bayesian spatio-temporal modelling. Spatial and spatio-temporal epidemiology. 2020 Jun 1;33:100335.
8	Aswi A, Cramb S, Duncan E, Mengersen K. Evaluating the impact of a small number of areas on spatial estimation. International journal of health geographics. 2020 Dec;19(1):1-4.
9	Baker J, White N, Mengersen K, Rolfe M, Morgan GG. Joint modelling of potentially avoidable hospitalisation for five diseases accounting for spatiotemporal effects: A case study in New South Wales, Australia. PLoS One. 2017 Aug 30;12(8):e0183653.
10	Blain AP, Thomas MF, Shirley MD, Simmister C, Elemraid MA, Gorton R, Pearce MS, Clark JE, Rushton SP, Spencer DA. Spatial variation in the risk of hospitalization with childhood pneumonia and empyema in the North of England. Epidemiology & Infection. 2014 Feb;142(2):388-98.
11	Chou MP, Clements AC, Thomson RM. A spatial epidemiological analysis of nontuberculous mycobacterial infections in Queensland, Australia. BMC Infectious Diseases. 2014 Dec;14(1):1-0.
12	Cramb SM, Baade PD, White NM, Ryan LM, Mengersen KL. Inferring lung cancer risk factor patterns through joint Bayesian spatio-temporal analysis. Cancer Epidemiology. 2015 Jun 1;39(3):430-9.
13	Danwang C, Khalil É, Achu D, Ateba M, Abomabo M, Souopgui J, De Keukeleire M, Robert A. Fine scale analysis of malaria incidence in under-5: hierarchical Bayesian spatio-temporal modelling of routinely collected malaria data between 2012–2018 in Cameroon. Scientific reports. 2021 Jun 1;11(1):1-0.
14	Darikwa TB, Manda SO. Spatial co-clustering of cardiovascular diseases and select risk factors among adults in South Africa. International Journal of Environmental Research and Public Health. 2020 Jan;17(10):3583.
15	Desjardins MR, Eastin MD, Paul R, Casas I, Delmelle EM. Space–Time Conditional Autoregressive Modeling to Estimate Neighborhood-Level Risks for Dengue Fever in Cali, Colombia. The American journal of tropical medicine and hygiene. 2020 Nov;103(5):2040.
16	Dhewantara PW, Marina R, Puspita T, Ariati Y, Purwanto E, Hananto M, Hu W, Magalhaes RJ. Spatial and temporal variation of dengue incidence in the island of Bali, Indonesia: An ecological study. Travel medicine and infectious disease. 2019 Nov 1;32:101437.
17	Donkor E, Kelly M, Eliason C, Amotoh C, Gray DJ, Clements AC, Wangdi K. A bayesian spatio-temporal analysis of malaria in the greater Accra region of Ghana from 2015 to 2019. International journal of environmental research and public health. 2021 Jun 4;18(11):6080.

18	Feng CX. Bayesian joint modeling of correlated counts data with application to adverse birth outcomes. Journal of Applied Statistics. 2015 Jun 3;42(6):1206-22.
19	Gelaw YA, Magalhães RJ, Assefa Y, Williams G. Spatial clustering and socio-demographic determinants of HIV infection in Ethiopia, 2015–2017. International Journal of Infectious Diseases. 2019 May 1;82:33-9.
20	Hanandita W, Tampubolon G. Geography and social distribution of malaria in Indonesian Papua: a cross-sectional study. International journal of health geographics. 2016 Dec;15(1):1-5.
21	Hu W, Clements A, Williams G, Tong S, Mengersen K. Spatial patterns and socioecological drivers of dengue fever transmission in Queensland, Australia. Environmental health perspectives. 2012 Feb;120(2):260-6.
22	Huang X, Lambert S, Lau C, Magalhaes RS, Marquess J, Rajmokan M, Milinovich G, Hu W. Assessing the social and environmental determinants of pertussis epidemics in Queensland, Australia: a Bayesian spatio-temporal analysis. Epidemiology & Infection. 2017 Apr;145(6):1221-30.
23	Ibeji JU, Mwambi H, Iddrisu AK. Spatial variation and risk factors of malaria and anaemia among children aged 0 to 59 months: a cross-sectional study of 2010 and 2015 datasets. Scientific Reports. 2022 Jul 7;12(1):1-5.
24	Jürgens V, Ess S, Phuleria HC, Früh M, Schwenkglenks M, Frick H, Cerny T, Vounatsou P. Bayesian spatio-temporal modelling of tobacco-related cancer mortality in Switzerland. Geospatial Health. 2013;7(2):219-36.
25	Kandhasamy C, Ghosh K. Relative risk for HIV in India–An estimate using conditional auto-regressive models with Bayesian approach. Spatial and spatio-temporal epidemiology. 2017 Feb 1;20:27-34.
26	Kigozi SP, Kigozi RN, Sebuguzi CM, Cano J, Rutazaana D, Opigo J, Bousema T, Yeka A, Gasasira A, Sartorius B, Pullan RL. Spatial-temporal patterns of malaria incidence in Uganda using HMIS data from 2015 to 2019. BMC public health. 2020 Dec;20(1):1-4.
27	Lal A, Swaminathan A, Holani T. Spatial clusters of Clostridium difficile infection and an association with neighbourhood socio-economic disadvantage in the Australian Capital Territory, 2004–2014. Infection, Disease & Health. 2020 Feb 1;25(1):3-10.
28	Law J. Exploring the specifications of spatial adjacencies and weights in Bayesian spatial modeling with intrinsic conditional autoregressive priors in a small-area study of fall injuries. AIMS public health. 2016;3(1):65.
29	Li M, Baffour B, Richardson A. Bayesian spatial modelling of early childhood development in Australian regions. International journal of health geographics. 2020 Dec;19(1):1-6.
30	Lome-Hurtado A, Lartigue-Mendoza J, Trujillo JC. Modelling local patterns of child mortality risk: a Bayesian Spatio-temporal analysis. BMC public health. 2021 Dec;21(1):1-2.
31	Lome-Hurtado A, Li G, Touza-Montero J, White PC. Patterns of low birth weight in greater Mexico City: A Bayesian spatio-temporal analysis. Applied Geography. 2021 Sep 1;134:102521.
32	Lubinda J, Bi Y, Hamainza B, Haque U, Moore AJ. Modelling of malaria risk, rates, and trends: A spatiotemporal approach for identifying and targeting sub-national areas of high and low burden. PLoS computational biology. 2021 Mar 1;17(3):e1008669.
33	Ngwira A. Shared geographic spatial risk of childhood undernutrition in Malawi: An application of joint spatial component model. Public Health in Practice. 2022 Jun 1;3:100224.
34	Ntirampeba D, Neema I, Kazembe L. Modelling spatio-temporal patterns of disease for spatially misaligned data: An application on measles incidence data in Namibia from 2005-2014. PLoS One. 2018 Aug 13;13(8):e0201700.
35	Odhiambo JN, Sartorius B. Mapping of anaemia prevalence among pregnant women in Kenya (2016–2019). BMC pregnancy and childbirth. 2020 Dec;20(1):1-1.
36	Ogunsakin RE, Ginindza TG. Bayesian Spatial Modeling of Diabetes and Hypertension: Results from the South Africa General Household Survey. International Journal of Environmental Research and Public Health. 2022 Jul 22;19(15):8886.

37	Okango E, Mwambi H, Ngesa O. Spatial modeling of HIV and HSV-2 among women in Kenya with spatially varying coefficients. BMC public health. 2016 Dec;16(1):1-3.
38	Okango E, Mwambi H, Ngesa O, Achia T. Semi-parametric spatial joint modeling of HIV and HSV-2 among women in Kenya. PloS one. 2015 Aug 10;10(8):e0135212.
39	Okunlola OA, Oyeyemi OT, Lukman AF. Modeling the relationship between malaria prevalence and insecticide-treated bed net coverage in Nigeria using a Bayesian spatial generalized linear mixed model with a Leroux prior. Epidemiology and Health. 2021;43.
40	Qi X, Hu W, Mengersen K, Tong S. Socio-environmental drivers and suicide in Australia: Bayesian spatial analysis. BMC public health. 2014 Dec;14(1):1-0.
41	Raei M, Schmid VJ, Mahaki B. Bivariate spatiotemporal disease mapping of cancer of the breast and cervix uteri among Iranian women. Geospatial Health. 2018 May 8;13(1).
42	Reid HL, Haque U, Roy S, Islam N, Clements AC. Characterizing the spatial and temporal variation of malaria incidence in Bangladesh, 2007. Malaria Journal. 2012 Dec;11(1):1-8.
43	Roza DL, Caccia-Bava MD, Martinez EZ. Spatio-temporal patterns of tuberculosis incidence in Ribeirão Preto, State of São Paulo, southeast Brazil, and their relationship with social vulnerability: a Bayesian analysis. Revista da Sociedade Brasileira de Medicina Tropical. 2012;45:607-15.
44	Saijo Y, Yoshioka E, Kawanishi Y, Nakagi Y, Hanley SJ, Yoshida T. Relationships between road-distance to primary care facilities and ischemic heart disease and stroke mortality in Hokkaido, Japan: A Bayesian hierarchical approach to ecological count data. Journal of general and family medicine. 2018 Jan;19(1):4-8.
45	Sharafi Z, Asmarian N, Hoorang S, Mousavi A. Bayesian spatio-temporal analysis of stomach cancer incidence in Iran, 2003–2010. Stochastic environmental research and risk assessment. 2018 Oct;32(10):2943-50.
46	Thiam S, Cissé G, Stensgaard AS, Niang-Diène A, Utzinger J, Vounatsou P. Bayesian conditional autoregressive models to assess spatial patterns of diarrhoea risk among children under the age of 5 years in Mbour, Senegal. Geospatial Health. 2019 Nov 6;14(2).
47	Tsheten T, Clements AC, Gray DJ, Wangchuk S, Wangdi K. Spatial and temporal patterns of dengue incidence in Bhutan: a Bayesian analysis. Emerging Microbes & Infections. 2020 Jan 1;9(1):1360-71.
48	Wangdi K, Clements AC. Spatial and temporal patterns of diarrhoea in Bhutan 2003–2013. BMC infectious diseases. 2017 Dec;17(1):1-9.
49	Wangdi K, Clements AC, Du T, Nery SV. Spatial and temporal patterns of dengue infections in Timor-Leste, 2005–2013. Parasites & vectors. 2018 Dec;11(1):1-9.
50	Wangdi K, Wetzler E, Cox H, Marchesini P, Villegas L, Canavati S. Spatial patterns and climate drivers of malaria in three border areas of Brazil, Venezuela and Guyana, 2016-2018.
51	Wangdi K, Xu Z, Suwannatrai AT, Kurscheid J, Lal A, Namgay R, Glass K, Gray DJ, Clements AC. A spatio-temporal analysis to identify the drivers of malaria transmission in Bhutan. Scientific reports. 2020 Apr 27;10(1):1-0.
52	Xu Z, Hu W, Tong S. The geographical co-distribution and socio-ecological drivers of childhood pneumonia and diarrhoea in Queensland, Australia. Epidemiology & Infection. 2015 Apr;143(5):1096-104.
